# Mitochondrial fusion protein: a new therapeutic target for lung injury diseases

**DOI:** 10.3389/fphys.2025.1500247

**Published:** 2025-03-19

**Authors:** Guiyang Jia, Erqin Song, Qianxia Huang, Miao Chen, Guoyue Liu

**Affiliations:** ^1^ Department of Critical Care Medicine, The Second Affiliated Hospital of Zunyi Medical University, Zunyi, China; ^2^ Department of Critical Care Medicine, Affiliated Hospital of Zunyi Medical University, Zunyi, China

**Keywords:** mitochondria, lung injury, mitochondrial fusion, mitofusin 1/2, optic atrophy 1

## Abstract

Mitochondria are essential organelles responsible for cellular energy supply. The maintenance of mitochondrial structure and function relies heavily on quality control systems, including biogenesis, fission, and fusion. Mitochondrial fusion refers to the interconnection of two similar mitochondria, facilitating the exchange of mitochondrial DNA, metabolic substrates, proteins, and other components. This process is crucial for rescuing damaged mitochondria and maintaining their normal function. In mammals, mitochondrial fusion involves two sequential steps: outer membrane fusion, regulated by mitofusin 1 and 2 (MFN1/2), and inner membrane fusion, mediated by optic atrophy 1 (OPA1). Dysfunction in mitochondrial fusion has been implicated in the development of various acute and chronic lung injuries. Regulating mitochondrial fusion, maintaining mitochondrial dynamics, and improving mitochondrial function are effective strategies for mitigating lung tissue and cellular damage. This study reviews the expression and regulatory mechanisms of mitochondrial fusion proteins in lung injuries of different etiologies, explores their relationship with lung injury diseases, and offers a theoretical foundation for developing novel therapeutic approaches targeting mitochondrial fusion proteins in lung injury.

## 1 Introduction

Mitochondria are crucial organelles in the cell that play a crucial role in the energy supply of cells. They are also the main source of reactive oxygen species, and their dysfunction leads to cell damage, contributing considerably to the development of multiple lung diseases (Tulen et al., 2023; Pokharel et al., 2024). The maintenance of mitochondrial structure and function relies on quality control systems, such as mitochondrial biogenesis, fission, and fusion (Atici et al., 2023). The dynamic balance between fission and fusion regulates mitochondrial size, number, and shape. Disruptions in this balance, such as excessive fission or reduced fusion, result in mitochondrial fragmentation and impaired function (Chan, 2020). Mitochondrial fission generates two functional submitochondria through symmetrical division, or separates damaged components via asymmetrical division, producing one healthy and one dysfunctional fragment to preserve normal mitochondrial activity (Quiles and Gustafsson, 2022). Conversely, mitochondrial fusion connects adjacent mitochondria, allowing material exchange, repair of damaged mitochondria, and restoration of normal function (Yapa et al., 2021).

Lung injury encompasses acute and chronic damage to lung tissues and cells caused by factors such as infection, cigarette smoke (CS), particulate matter (PM), hyperoxia, hypoxia, and trauma. These conditions disrupt gas exchange in the lungs, potentially leading to acute or chronic respiratory failure, which can be life-threatening (Bos and Ware, 2022; Wick and Matthay, 2021). Mitochondrial fusion and fission play key roles in maintaining mitochondrial homeostasis and are implicated in the onset and progression of lung injuries from various causes. Regulating mitochondrial function has emerged as a promising therapeutic strategy to alleviate lung tissue and cellular damage (Ye et al., 2024; Zhan and Shen, 2022). This study reviews research on mitochondrial fusion proteins in various lung injury conditions, examines their association with lung diseases, and offers insights and new perspectives on therapeutic strategies and future research targeting these proteins in lung injury.

## 2 Mitochondrial fusion protein and lung injury

**FIGURE 1:**
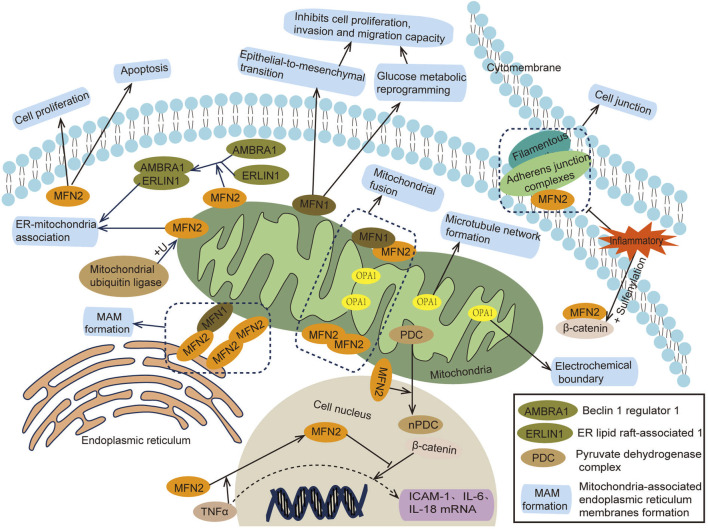
Mechanism of action of mitochondrial fusion protein.

Mammalian mitochondria are double-membrane organelles, and their fusion occurs in two steps: outer membrane fusion and inner membrane fusion. Outer membrane fusion is primarily regulated by mitofusin 1 and 2 (MFN1/2) (Zorzano et al., 2010), while inner membrane fusion is regulated by optic atrophy 1 (OPA1) (Gao and Hu, 2021). MFN1 and MFN2 are GTPase proteins. MFN1 contains GTPase domains and a four-helix bundle, enabling mitochondrial outer membrane fusion through synergistic oligomerization and GTPase hydrolysis of adjacent mitochondria (Cao et al., 2017; Alghamdi, 2024). Additionally, MFN1 inhibits cell proliferation, migration, and invasion (Zhang et al., 2020). MFN2 can form MFN1/MFN2 or MFN2/MFN2 polymers, facilitating mitochondrial proximity, perinuclear aggregation, and interactions with the endoplasmic reticulum (ER) (Mao et al., 2022; Zervopoulos et al., 2022). Beyond its mitochondrial functions, MFN2 can also suppress inflammation by stabilizing cell–cell adhesion and binding to the transcription activator β-catenin (Kim et al., 2021). Moreover, MFN2 plays a role in cell proliferation, apoptosis, and autophagy, contributing to fibroproliferative diseases (such as pulmonary fibrosis, cirrhosis, and cardiovascular fibrosis), atherosclerosis, pulmonary hypertension, tumors and other diseases (Xin et al., 2021). OPA1, located in the inner mitochondrial membrane, collaborates with MFN1 and MFN2 to regulate mitochondrial fusion (Cipolat et al., 2004). OPA1 also interacts with mitochondrial cristae regulators, the mitochondrial contact site and cristae organizing system (MICOS) protein family, and the protease Yme1L to modulate the shape and tightness of mitochondrial cristae (Hu et al., 2020). These interactions maintain the electrochemical boundary between cristae and the inner boundary membrane, preventing localized cristae dysfunction from affecting the entire mitochondria, thereby preserving mitochondrial membrane potential and cellular respiratory function (Wolf et al., 2019). A deficiency in OPA1 leads to mitochondrial fragmentation, translocation and microtubule destruction, impaired degranulation, disrupted functional network formation, and ultimately mitochondrial dysfunction (Amini et al., 2018) (Figure 1).

Mitochondrial fusion facilitates the exchange of mitochondrial DNA (mtDNA), metabolic substrates, and proteins, while also regulating mtDNA replication (Silva Ramos et al., 2019). Moreover, mtDNA plays a role in modulating inflammation and immune responses. Dysfunctional mitochondrial fusion contributes to the development of various acute and chronic lung injuries (Yue and Yao, 2016). Regulating mitochondrial fusion, maintaining mitochondrial dynamics, improving mitochondrial function, and reducing lung tissue and cell damage are effective strategies for treating lung injury (Sun et al., 2023). Studies have shown that factors such as infection (Yu et al., 2016a), cigarette smoke (CS) (Wang et al., 2020), environmental changes, hyperoxia or hypoxia (Ma et al., 2017), drugs, and toxins can impact mitochondrial fusion proteins, leading to lung injury.

## 3 Mitochondrial fusion proteins in lung injury caused by different factors

### 3.1 Infection

Infection and sepsis, resulting from abnormal immune responses, are major contributors to acute lung injury. Mitochondrial dysfunction, caused by the abnormal expression of mitochondrial fusion proteins, is one of its pathogenesis (Mohsin et al., 2021; Oliveira et al., 2021). Investigating and regulating these proteins is effective for preventing and treating infection-induced lung injury.

**FIGURE 2:**
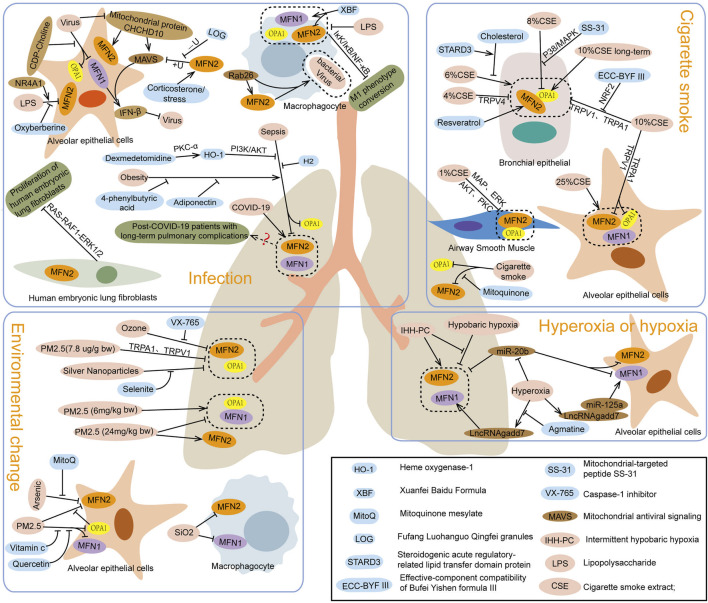
Effects of infection, cigarette smoke, environmental factors, and hyperoxia or hypoxia on mitochondrial fusion protein expression.

Studies have shown that patients recovering from COVID-19 may experience long-term pulmonary complications, including dyspnea, impaired lung function, and pulmonary fibrosis (Desai et al., 2022). Serum MFN2 levels in patients with such complications were significantly higher than in those without pulmonary issues, indicating that MFN2, a mitochondrial fusion protein, may contribute to the development of these complications after COVID-19 infection (Siekacz et al., 2023). Similarly, animal and cell experiments have demonstrated that infection with influenza A virus or Newcastle disease virus alters the expression and protein levels of MFN1 and OPA1 in alveolar epithelial cells. These changes impair mitochondrial fusion, leading to mitochondrial fragmentation and dysfunction (Doolittle et al., 2022; Yu et al., 2024). Moreover, experiments targeting lung macrophages revealed that Rab26 promotes MFN2 expression and interacts with it to regulate macrophage phagocytosis and bacterial clearance, thereby alleviating acute respiratory distress syndrome in mice (Wu et al., 2023). Additionally, stress or corticosterone-induced upregulation of MFN2 expression in mouse lung tissue was found to suppress MAVS and β-interferon (IFN-β) levels by recruiting E3 ligase SYVN1, initiating ubiquitination and degradation of mitochondrial antiviral signaling (MAVS) protein. This process blocks the MAVS/IFN-β response and enhances influenza A virus replication in cells (Luo et al., 2020). Notably, Fufang Luohanguo Qingfei granules (LQG) were observed to inhibit MFN2-mediated MAVS ubiquitination in mouse lung tissue, enhance the IFN-β antiviral response, and reduce the susceptibility of mice to influenza A virus (Lu et al., 2024). In summary, viral or bacterial infections can lead to lung injury through distinct mechanisms involving mitochondrial fusion proteins. On one hand, decreased levels of MFN1, MFN2, and OPA1 disrupt mitochondrial fusion, causing fragmentation and dysfunction. This reduction in MFN2 also impairs macrophage phagocytosis and bacterial clearance, exacerbating lung tissue and cell damage. On the other hand, MFN1 and MFN2 can promote viral replication and worsen lung injury by modulating MAVS pathways (Figure 2).

Studies on sepsis-induced lung injury, caused by cecal ligation puncture or lipopolysaccharide (LPS), have shown decreased expression levels of mitochondrial fusion proteins MFN2 and OPA1 in lung tissue, alveolar epithelial cells, and macrophages (Li Z. et al., 2023; Shi et al., 2019). Dexmedetomidine treatment suppressed the upregulation of MFN1, MFN2, and OPA1 in LPS-induced lung tissue injury in mice (Song et al., 2022), while Hydrogen inhalation inhibited the downregulation of MFN2 in cecal ligation puncture-induced lung injury, thereby alleviating damage (Dong et al., 2018). Similarly, heme oxygenase-1 treatment increased MFN1 expression in lung tissue and macrophages (Yu et al., 2016b), whereas oxyberberine reduced MFN2 expression in alveolar epithelial cells exposed to LPS, mitigating cell injury (Zhao et al., 2021). In contrast, NR4A1 inhibited MFN1, MFN2, and OPA1 expression, worsening lung injury caused by sepsis (Zhu et al., 2022). Additionally, obesity-induced MFN2 downregulation exacerbates LPS-induced lung injury via ER stress. Rosiglitazone, a peroxisome proliferator-activated receptor-γ activator, was found to promote MFN2 expression, inhibit ER stress, enhance mitochondrial biogenesis, and alleviate lung injury (Wei et al., 2020; Tang et al., 2022). Further research revealed that MFN2 deletion increases pulmonary vascular permeability by impairing platelet number and function, intensifying LPS-induced lung injury and inflammatory bleeding (Jacob et al., 2024). Besides, MFN2 overexpression in fibroblasts promotes apoptosis, reduces activity, and inhibits proliferation and collagen synthesis, potentially limiting pulmonary fibrosis (Li et al., 2020). In summary, reduced levels of MFN1, MFN2, and OPA1 contribute to lung injury by disrupting alveolar epithelial cells, decreasing surfactant production, and impairing macrophage-mediated immune responses. The absence of MFN2 also increases vascular permeability and promotes fibroblast proliferation and collagen synthesis, contributing to pulmonary fibrosis. Thus, regulating or enhancing mitochondrial fusion protein activity represents a promising therapeutic target for sepsis-induced lung injury and pulmonary fibrosis (Figure 2).

### 3.2 Cigarette smoke (CS)

China is one of the largest producers and consumers of cigarettes. Over the past 2 decades, rising cigarette consumption, combined with an aging population, is projected to significantly increase smoking-related deaths in the coming decades (Chan et al., 2023). Research shows that cigarette smoke (CS) induces neutrophil infiltration and mitochondrial dysfunction in lung tissue, leading to excessive reactive oxygen species production, cell aging, DNA damage, and chronic obstructive pulmonary disease (COPD) (Antunes et al., 2021). Mitochondrial oxidative stress and dysfunction, driven by abnormal expression of mitochondrial fusion proteins, play a central role in this process (Hara et al., 2013; Yang D. et al., 2021). Understanding and regulating mitochondrial fusion proteins hold significant meaning for mitigating CS-induced lung injury, improving public health, and reducing the associated economic burden.

After exposure to cigarette smoke (CS), the expressions of mitochondrial fusion proteins MFN1, MFN2, and OPA1 in lung tissue were found to decrease (Maremanda et al., 2019). Pretreatment with the mitochondrial-targeted antioxidant SS-31, however, elevated OPA1 levels, alleviating lung injury caused by CS (Yang DQ. et al., 2021). Studies in cell models showed that CS exposure reduced MFN2 expression in airway smooth muscle and epithelial cells (Aravamudan et al., 2017; Li L. et al., 2022), as well as both OPA1 and MFN2 in alveolar and bronchial epithelial cells (Wang et al., 2019), exacerbating cellular aging and damage. Silencing MFN1, MFN2, or OPA1 induced mitochondrial fragmentation, increased reactive oxygen species production, and aggravated bronchial epithelial cell senescence (Hara et al., 2013). Various therapeutic interventions demonstrated protective effects: Bufei Yichen formula inhibited the reduction in MFN2 expression in bronchial epithelial cells exposed to CS extract (CSE) (Li MY. et al., 2022); transient receptor potential channel antagonists upregulated MFN2 and OPA1 expression in bronchial epithelial cells (Rao et al., 2021); and resveratrol reversed CSE-induced downregulation of MFN2 (Song et al., 2017). Promoters of MFN2 (leflunomide) or OPA1 (BGP-15) increased their respective protein levels in alveolar epithelial cells, stabilizing mitochondrial morphology and mitigating cell damage (Li C. et al., 2023). Interestingly, low-dose CS exposure was found by Allweg et al. to elevate MFN2 levels in alveolar epithelial cell mitochondria, leading to excessive mitochondrial fusion (allweg et al., 2014). Similarly, Hoffmann et al. observed that long-term CS exposure increased OPA1 levels in bronchial epithelial cells, highlighting the dependence of CS effects on concentration, dose, exposure duration, and individual metabolic conditions (Hoffmann et al., 2013; Aravamudan et al., 2014). Furthermore, OPA1 exists in multiple isoforms that jointly regulate mitochondrial morphology and crista integrity, and disruptions in their balance can impair mitochondrial function (Del Dotto et al., 2018). CS exposure was also shown to alter the quantity and ratio of OPA1 isoforms in bronchial epithelial cells, fibroblasts, and embryonic fibroblasts (Maremanda et al., 2021), leading to abnormal mitochondrial morphology and function. Despite these findings, the mechanisms underlying the regulation of OPA1 isoforms and mitochondrial functional improvement require further investigation.

### 3.3 Environmental change

Environmental pollutants such as particulate matter (PM), organic compounds, and metals can enter the human body through various pathways, leading to an increase in reactive oxygen species (ROS), mitochondrial dysfunction, impaired ATP production, and disrupted cellular homeostasis, ultimately contributing to the development of numerous diseases (Manzano-Covarrubias et al., 2023). As air pollution levels continue to rise, the susceptibility to respiratory illnesses, along with the incidence and mortality of chronic cardiovascular and pulmonary conditions, is expected to grow. According to the World Health Organization, over seven million deaths are attributed to air pollution each year (Monoson et al., 2023). In cases of lung injury induced by environmental factors, abnormal expression of mitochondrial fusion proteins, such as MFN2 and OPA1, in bronchial epithelial cells has been shown to exacerbate lung tissue damage and fibrosis by impairing mitochondrial and endoplasmic reticulum (ER) function (Bhat et al., 2022; Huang et al., 2020). Modulating the expression of these mitochondrial fusion proteins, particularly MFN1 and MFN2, can influence the mitochondrial morphology and functionality of alveolar epithelial cells, as well as lipid metabolism and surfactant production (Chung et al., 2019). This regulation presents a promising strategy for mitigating lung injury and pulmonary fibrosis caused by environmental pollutants.

In studies of lung injury caused by PM2.5 intranasal infusion and ozone inhalation, decreased expressions of OPA1 and MFN2 were observed in mouse lung tissue (Xu et al., 2021; Weng et al., 2024). However, the use of caspase-1 inhibitor VX765 inhibited the ozone-induced reduction of MFN2 expression (Xu et al., 2019a), while transient receptor potential anchoring protein-1 inhibitor A967079 or TRPV1 inhibitor AMG9810 mitigated the reduction of MFN2 and OPA1 expression (Li C. et al., 2022; Xu et al., 2019b), alleviating oxidative stress and airway inflammation in lung tissue. In rat models with lung injury induced by silver nanoparticles, OPA1 and MFN2 expressions were similarly decreased, but treatment with selenite inhibited this decline, reducing mitochondrial damage and improving lung tissue pathology and ultrastructure (Ma et al., 2020). Cell studies have demonstrated that arsenic exposure reduces MFN2 expression in alveolar epithelial cells, leading to ER dysfunction and the onset of ferroptosis. However, preconditioning with mitoquinone mesylate to prevent the decline of MFN2 expression or directly overexpressing MFN2 was shown to improve ER function and mitigate arsenic-induced acute lung injury (Li MD. et al., 2022). PM2.5 exposure significantly reduced MFN2 and OPA1 levels in alveolar epithelial cells and MFN1 levels in bronchial epithelial cells. Pretreatment with the OPA1 promoter BGP-15 effectively increased OPA1 and MFN2 levels (Liu Q. et al., 2023). Interestingly, low doses of PM (1.5 and 6 mg/kg body weight) were reported to increase MFN1 and OPA1 expressions in alveolar epithelial cells, while higher doses (24 mg/kg body weight) caused a decrease in MFN1, MFN2, and OPA1 expressions, leading to increased mitochondrial fragmentation (Li et al., 2015). These findings suggest that mitochondrial fusion protein expression varies with PM concentration and dose. Low doses of PM may induce mitochondrial damage and increase fusion protein levels, promoting mitochondrial repair, while high doses overwhelm the repair mechanisms, reducing fusion proteins and causing greater damage. Additionally, low-dose PM may increase mitochondrial fusion proteins but disrupt the fusion–fission balance, mitochondrial-ER connections, and mitochondrial perinuclear aggregation, contributing to mitochondrial dysfunction. In summary, mitochondrial fusion proteins such as MFN2 and OPA1 play dual roles in environmental factor-induced lung injury. On one hand, they alleviate oxidative stress and airway inflammation and reduce harmful particle accumulation in mitochondria. On the other hand, their dysregulation under specific conditions exacerbates lung damage. Furthermore, alterations in MFN1 and MFN2 expression in alveolar macrophages are linked to lung injury caused by environmental factors (Zhang et al., 2019), with macrophage and fibroblast dysfunction playing key roles in airway inflammation and injury (Li CH. et al., 2022; Liu et al., 2024). Additional research is needed to clarify the mechanisms through which mitochondrial fusion proteins regulate alveolar macrophage functions and their role in mitigating lung injury. The involvement of MFN1 in environmental factor-induced lung injury also requires further exploration to understand its specific changes and mechanisms.

### 3.4 Hyperoxia or hypoxia

Oxygen is essential for maintaining normal human metabolism as well as organ and cellular functions. However, prolonged exposure to either Hyperoxia (Amarelle et al., 2021) or hypoxia (Mikami et al., 2023) can increase reactive oxygen species production, disrupt the oxidative and antioxidant balance, and impair mitochondrial quality control processes, including mitochondrial biogenesis, fission, and fusion (Bartman et al., 2021). These changes can exacerbate pre-existing lung injuries or lead to new lung damage. Regulating mitochondrial quality control processes offers a potential therapeutic strategy to address such cellular dysfunctions (Ma et al., 2023).

In hypoxia-induced lung injury, MFN2 expression is reduced, while changes in MFN1 and OPA1 levels remain insignificant. Notably, pretreatment with dexamethasone to restore MFN2 levels (Chitra and Boopathy, 2013) or intermittent hypobaric hypoxia to upregulate compensatory expression of MFN1 and MFN2 (Chitra and Boopathy, 2014) has been shown to enhance hypoxia tolerance in experimental animals. In contrast, hyperoxia leads to an increase in MFN1 levels in alveolar epithelial cells, contributing to cellular damage (Mu et al., 2021). Treatment with agmatine or overexpression of miR-20b, both of which negatively regulate MFN1 expression, alleviates alveolar epithelial cell apoptosis (Liu G. et al., 2023; Liu et al., 2019). Additionally, hyperoxia-induced injury decreases MFN1, MFN2, and OPA1 expression in lung mesenchymal stem cells, but cinnamaldehyde treatment has been found to upregulate these proteins, mitigate oxidative stress, and reduce apoptosis (Ke et al., 2022) (Figure 2). Hyperoxia and hypoxia both influence mitochondrial fusion protein expression in alveolar epithelial cells, lung mesenchymal stem cells, and lung tissue, resulting in mitochondrial dysfunction, increased apoptosis, and exacerbated lung injury. Most studies indicate that mitochondrial fusion proteins support normal mitochondrial function by maintaining fusion. Conversely, reduced expression of these proteins leads to impaired fusion, mitochondrial dysfunction, cellular damage, and tissue injury. However, the mechanisms by which elevated mitochondrial fusion protein levels contribute to tissue damage remain unclear. We propose several possible mechanisms: (1) Mitochondrial fusion and fission are interrelated processes. Increased fusion protein expression may activate mitochondrial fission, creating an imbalance where fission dominates fusion, leading to mitochondrial fragmentation and dysfunction. (2) Excessive fusion protein levels may disrupt the balance of mitochondrial fusion and fission, causing abnormalities in mitochondrial networks and metabolic dysfunction. This imbalance reduces cellular adaptability and makes cells more susceptible to damage. (3) Beyond its role in fusion, mitochondrial fusion proteins are involved in other cellular processes, and their elevation may exacerbate tissue damage through these pathways. (4) Excessive fusion in the context of insufficient mitochondrial biogenesis may also lead to mitochondrial dysfunction (Yoon et al., 2006). This could be due to inadequate biogenesis and fission following cellular injury, where excessive fusion reduces mitochondrial numbers, impairs respiratory activity, increases intracellular reactive oxygen species, and aggravates tissue damage.

### 3.5 Other factors

In addition to the previously mentioned factors, poisoning, drug exposure, mechanical ventilation, and inflammation originating from other tissues and organs can also contribute to lung injury Table 1. In paraquat-induced lung injury, expressions of OPA1 and MFN2 are significantly reduced. However, treatment with oxaloacetate acid increases the levels of these proteins, alleviating lung damage (Li W. et al., 2022). Similarly, mitochondria-targeted antioxidants can promote MFN1/MFN2-mediated mitochondrial fusion, reducing paraquat-induced lung injury and extending the survival time of mice (Liu et al., 2022). In cases of lung injury caused by chemotherapy drugs, MFN1 and MFN2 expression levels are decreased, but treatment with Pleurotus eryngii extract can restore these levels and mitigate lung tissue damage (Tektemur et al., 2023). In lung injury induced by mechanical ventilation, MFN1 expression in lung tissues is upregulated, possibly due to oxygen oversupply (Lin et al., 2018; Xu et al., 2023). However, excessive oxygen has been shown to induce MFN1 upregulation in cells, exacerbating lung injury (Mu et al., 2021). Lung injury from mechanical ventilation is also associated with high airway pressures caused by increased tidal volume, though whether airway pressure specifically alters mitochondrial fusion proteins remains unclear. Additionally, MFN2 levels in lung tissues are elevated after chronic cold exposure, which enhances mitochondrial fusion but may disrupt mitochondrial physiological functions (Teng et al., 2022). In pulmonary inflammation caused by nonalcoholic fatty liver disease, MFN1 expression is significantly reduced; however, exercise training can restore MFN1 levels and reestablish pulmonary mitochondrial homeostasis (Cho et al., 2022). TNF-α stimulation has been found to decrease MFN2 expression, promote ER stress, disrupt the mitochondrial-ER tether, fragment mitochondria, and increase mitochondrial biogenesis. These changes reduce oxygen utilization efficiency per mitochondrion while increasing overall oxygen consumption (Delmotte et al., 2021; Yap et al., 2020). It has been suggested that inflammation and pathological changes in other organs or tissues can influence the expression of mitochondrial fusion proteins in lung tissues, leading to lung tissue and cell damage. However, the specific mechanisms and regulatory pathways involved remain to be fully elucidated.

**TABLE 1:** Expression of mitochondrial fusion protein in lung injury caused by other causes.

Damage elements	*In vivo*or *in vitro*models	Protein changes	Intervention and effects	Reference
Paraquat	Human normal lung epithelial line BEAS-2B cells treated with Paraquat for 48 h	↓L-OPA1, ↓MFN2	Oxaloacetate acid: ↑ L-OPA1, ↑ MFN2 → Promoted mitochondrial fusion, alleviated lung injury, extended survival time	Li et al. (2022f)
Paraquat	Paraquat induced A549 cell injury model *in vitro*	↓MFN1, ↓MFN2	Mitoquinone alleviated paraquat-induced lung epithelial cell injury by enhancing mitochondrial fusion through MFN1 and MFN2 regulation	Liu et al. (2022)
Doxorubicin	The rats were given intraperitoneal injection of doxorubicin (10 mg/kg BW)	↓MFN1, ↓MFN2	Pleurotus eryngii extract restored the decreased expression levels of MFN1 and MFN2 induced by doxorubicin, thereby alleviating doxorubicin-induced lung injury	Tektemur et al. (2023)
High tidal volume	Tracheotomized rats underwent mechanical ventilation for 4 h	↑MFN1	The expression levels of mitofusin 1 in animals ventilated with HTV were significantly upregulated	Lin et al. (2018)
Chronic cold stress	Yorkshire pigs were exposed to natural ambient temperatures (7°C ± 3°C)	↑MFN2	Chronic cold stress induces high expression of MFN2, promoting mitochondrial fusion	Teng et al. (2022)
High-fat high-carbohydrate diet	Fed a high-fat, high-carbohydrate diet (58% fat, 25% carbohydrates, 17% protein) with drinking water containing 42 g/L carbohydrates in a high fructose- to-sucrose ratio	↓MFN1	A high-fat, high-carbohydrate diet impaired mitochondrial function and reduced MFN1 protein levels in rats, whereas aerobic exercise improved lung mitochondrial function by upregulating MFN1 levels	Cho et al. (2022)
Tumor necrosis factor-α (TNFα)	hASM cells were then exposed to TNFα for 12 h (50 ng/mL)	↓MFN2	TNFα reduced MFN2 expression in human airway smooth muscle (hASM) cells and stimulated their proliferation	Delmotte et al. (2021) Yap et al. (2020)

## 4 Summary and outlook

Mitochondrial fusion proteins play a critical role in maintaining normal cellular and tissue function, and their dysregulated expression has been implicated in the development of various diseases (Sharma et al., 2021). Investigating the relationship between mitochondrial fusion proteins and lung injury provides valuable insights for the prevention and treatment of lung injury-related conditions (Zacharioudakis and Gavathiotis, 2023). Currently, decreased expression of mitochondrial fusion proteins is believed to contribute to cellular and tissue dysfunction by impairing mitochondrial fusion and repair mechanisms (Gao and Hu, 2021). However, the mechanisms through which increased expression or other functions of mitochondrial fusion proteins exacerbate lung injury remain poorly understood. Studies have demonstrated that compounds such as dexmedetomidine, guanidine, cinnamon, vitamin C, quercetin, Fufang LQG, Bufei, and Yishen formula can modulate the expression of mitochondrial fusion proteins, reducing lung tissue and cellular damage. However, the majority of this research has been conducted at the animal and cellular levels, and the changes and mechanisms of mitochondrial fusion protein regulation in humans remain largely unexplored. Thus, further investigation into the role of mitochondrial fusion proteins and their modulation by related clinical drugs offers a promising approach to preventing and treating lung injury diseases.
